# Distinct Alterations in Oxygenation, Ion Composition and Acid-Base Balance in Cerebral Collaterals During Large-Vessel Occlusion Stroke

**DOI:** 10.1007/s00062-023-01296-w

**Published:** 2023-06-07

**Authors:** Jörn Feick, Mirko Pham, Alexander G. März, Marius L. Vogt, Marc Strinitz, Guido Stoll, Michael K. Schuhmann, Alexander M. Kollikowski

**Affiliations:** 1 Department of Neuroradiology, University Hospital Würzburg, Josef-Schneider-Straße 11, 97080 Würzburg, Germany; 2 Department of Neuroradiology, Klinikum rechts der Isar, Technical University Munich, Munich, Germany; 3 Department of Neurology, University Hospital Würzburg, Würzburg, Germany

**Keywords:** Acute ischemic stroke, Cerebral ischemia, Large-vessel occlusion, Arterial blood gas analysis, Endovascular thrombectomy

## Abstract

**Purpose:**

Disturbances of blood gas and ion homeostasis including regional hypoxia and massive sodium (Na^+^)/potassium (K^+^) shifts are a hallmark of experimental cerebral ischemia but have not been sufficiently investigated for their relevance in stroke patients.

**Methods:**

We report a prospective observational study on 366 stroke patients who underwent endovascular thrombectomy (EVT) for large-vessel occlusion (LVO) of the anterior circulation (18 December 2018–31 August 2020). Intraprocedural blood gas samples (1 ml) from within cerebral collateral arteries (ischemic) and matched systemic control samples were obtained according to a prespecified protocol in 51 patients.

**Results:**

We observed a significant reduction in cerebral oxygen partial pressure (−4.29%, *p*_a_O_2ischemic_ = 185.3 mm Hg vs. *p*_a_O_2systemic_ = 193.6 mm Hg; *p* = 0.035) and K^+^ concentrations (−5.49%, K^+^_ischemic_ = 3.44 mmol/L vs. K^+^_systemic_ = 3.64 mmol/L; *p* = 0.0083). The cerebral Na^+^:K^+^ ratio was significantly increased and negatively correlated with baseline tissue integrity (r = −0.32, *p* = 0.031). Correspondingly, cerebral Na^+^ concentrations were most strongly correlated with infarct progression after recanalization (r = 0.42, *p* = 0.0033). We found more alkaline cerebral pH values (+0.14%, pH_ischemic_ = 7.38 vs. pH_systemic_ = 7.37; *p* = 0.0019), with a time-dependent shift towards more acidotic conditions (r = −0.36, *p* = 0.055).

**Conclusion:**

These findings suggest that stroke-induced changes in oxygen supply, ion composition and acid-base balance occur and dynamically progress within penumbral areas during human cerebral ischemia and are related to acute tissue damage.

**Supplementary Information:**

The online version of this article (10.1007/s00062-023-01296-w) contains supplementary material, which is available to authorized users.

## Introduction

Despite major recent advances in endovascular stroke treatment [1], large-vessel occlusion (LVO) stroke remains a leading cause of death and acquired disability worldwide [2]. Hence, there is an urgent need to more clearly identify and better understand early pathophysiological events during cerebral ischemia not only in experimental settings but also in the human system [3, 4].

The cessation of cerebral blood flow (CBF) during LVO stroke results in a severe reduction of oxygen and glucose supply within the downstream vascular territory [5] and is accompanied by functionally relevant alterations of intracellular and extracellular ion composition in the affected brain region [6, 7]. These events are part of a complex set of mechanisms in response to the ischemic stimulus which has been coined the ischemic cascade. Key parenchymal and intravascular processes that parallel and/or define tissue damage include: bioenergetic failure, excitotoxicity, intracellular calcium (Ca^2+^) and sodium (Na^+^) overload, extracellular accumulation of potassium (K^+^), oxidative stress, and neuroinflammation [5–10]. These processes are closely interrelated as, e.g., severely reduced CBF leads to peri-infarct depolarization which is characterized by a regional switch from blood hypoxygenation to hyperoxygenation during propagation [11, 12]. In addition, there is evidence that also time-dependent regional pH shifts exist which likely indicate the differential fate of penumbral subareas [13]; however, available human data on regional blood gas and electrolyte alterations supporting the crucial role of blood oxygenation, acid-base balance and ion movements in LVO stroke are scarce and partly inconsistent [14–16]. Few groups including our own have established a sampling protocol for microcatheter-aspiration of cerebral blood samples from within the collateral circulation during LVO stroke immediately before therapeutic recanalization by endovascular thrombectomy (EVT) [14, 17, 18].

In this study, we now aim to retrace the aforementioned key experimental observations in a large and highly noise-controlled cohort where microcatheter aspiration attempts and analyses of cerebral samples were performed consecutively in every eligible anterior circulation LVO stroke patient. The biological relevance of changes in blood gas and ion composition was addressed by hypothesis-based association with 1) temporal, 2) clinical functional and 3) radiological structural parameters assessed during cerebral ischemia and in the clinical course after recanalization.

## Methods

### Study Design

We report a prospective observational single-center study conducted between 12 December 2018 and 31 August 2020, to investigate alterations in arterial blood gas (ABG) parameters within the collateral circulation (distal to the occlusion site) of LVO stroke patients. Endovascular sampling of ischemic blood from collateral blood vessels during the early phase of infarct formation was performed by microcatheter aspiration according to a prespecified protocol during emergency EVT [18–23]. Systemic arterial blood which was collected from the ipsilateral cervical internal carotid artery (ICA) under physiological flow conditions served as an intraindividual control.

### Inclusion and Exclusion Criteria

Inclusion criteria were defined as follows: (I) patients aged older than 18 years presenting with disabling first ever acute ischemic stroke which qualifies for EVT according to current guidelines and consensus recommendations [1, 24, 25] and (II) invasive angiographic confirmation of complete LVO of the following anterior circulation vessels: distal internal carotid artery (ICA-T), middle cerebral artery (MCA)-M1 segment or proximal MCA-M2 segment.

**Fig. 1:**
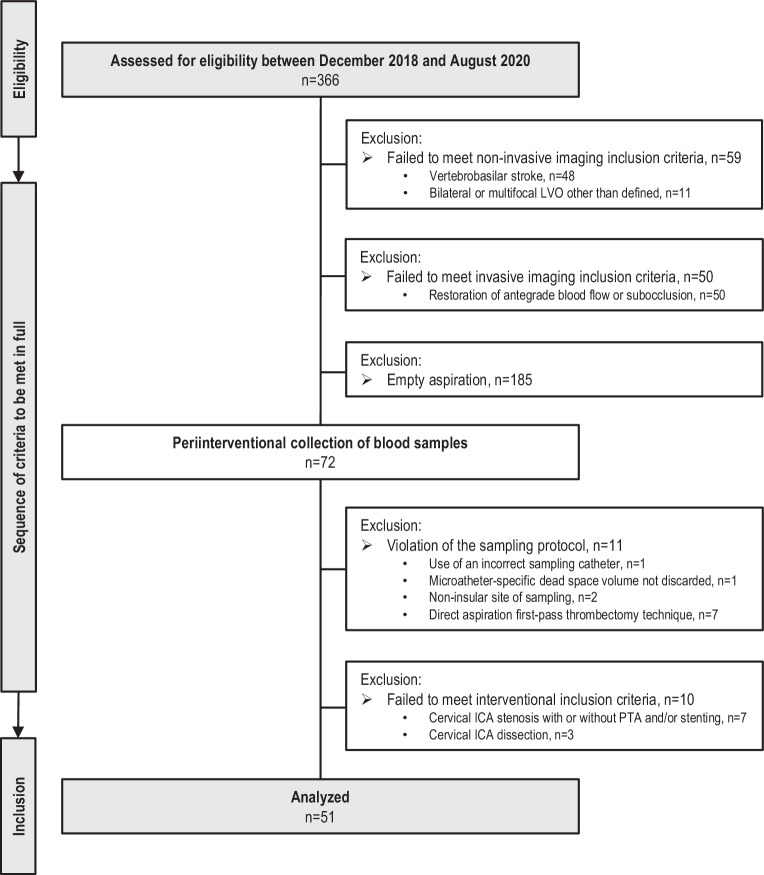
Flow chart of patient inclusion and exclusion. *CT* computed tomography, *ICA* internal carotid artery, *LVO* large-vessel occlusion, *PTA* percutaneous transluminal angioplasty

Patients were excluded based on the following criteria: (I) noninvasive or invasive angiographic confirmation of bilateral or multifocal vessel occlusions other than defined, (II) invasive angiographic confirmation of residual or restored antegrade CBF at the level of the EVT-qualifying lesion, (III) LVO in conjunction with either ≥ 50% cervical ICA stenosis or ICA dissection, (IV) intraprocedural percutaneous transluminal angioplasty (PTA) or stent implantation and (VI) principle deviations from the interventional protocol and sampling procedure [18–23]. The flow of patient inclusion and exclusion is presented in Fig. 1.

### Endovascular and Sampling Procedure

All endovascular treatments were performed by board-certified neurointerventionalists or supervised neurointerventional fellows on a biplane angiography system (Axiom Artis Q, Siemens Healthcare, Erlangen, Germany). Endovascular access for EVT was established by means of a transfemoral approach through the common femoral artery (CFA) using the modified Seldinger technique. According to our in-house standard of practice, pressurized flush systems (0.9% saline solution with 1 IU/ml unfractionated heparin) were used to avoid thrombus formation on the inner surface of catheters. A detailed description of the standard procedure of mechanical thrombectomy has been published previously [26]. Recanalization of the EVT-qualifying lesion by stent-embolus retrieval was preceded by microcatheter navigation (Neuroslider 27 or 21; Acandis, Pforzheim, Germany) into the mid-insular MCA-M2 segment. The following arterial blood samples were obtained by microcatheter aspiration: (1) distal to the occlusive lesion under persistent ischemic conditions and (2) under systemic physiological blood flow conditions at the cervical ICA level. Specifically, the first sample was obtained as follows: before device deployment and immediately after microcatheter positioning, the precise microcatheter dead space volume was aspirated with a 3 ml Luer lock syringe and discarded. Then, the target sample of 1 ml of ischemic blood from within the collateral circulation was obtained. The procedure of cerebral arterial blood sampling is displayed in supplemental Fig. 1. After termination of the therapeutic steps of EVT, systemic blood samples were obtained from cervical ICA level using the exact same technique as described for ischemic sample material.

### Processing of Blood Samples and Blood Gas Analyses

Arterial whole-blood samples were collected anaerobically in blood gas syringes containing Ca^2+^-balanced lithium heparin (S-Monovette, Sarstedt, Nümbrecht, Germany) and immediately analyzed (< 5 min) in a point-of-care blood gas analyzer (Rapidpoint 405, Siemens Healthcare Diagnostics, Erlangen, Germany). All syringes were carefully connected to the inlet of the analysis system without trapped air. A final sample volume of 200 µl was assessed for the following parameters: (1) pH, (2) arterial partial pressure of carbon dioxide (*p*_a_CO_2_), (3) arterial partial pressure of oxygen (*p*_a_O_2_), (4) base deviation in plasma (BE (B)), (5) standard bicarbonate (HCO_3_^−^ std), (6) oxygen saturation of hemoglobin (sO_2_), (7) Na^+^ ion concentration, (8) K^+^ ion concentration, (9) Na^+^:K^+^ ratio, (10) Ca^2+^ ion concentration, and (11) chloride (Cl^−^) ion concentration.

### Data Collection

Prospective data collection included (but was not limited to) the following demographic, clinical, radiological, interventional, and sampling-related variables: age, sex, cerebrovascular risk factors, baseline drug treatment, preprocedural blood pressure and heart rate, previous alteplase administration, National Institute of Health Stroke Scale (NIHSS) at admission and at 48 h, time of non-invasive and angiographic image acquisition, occlusion location, leptomeningeal collateral status, Alberta Stroke Program Early CT Score (ASPECTS) at baseline and at 48 h, time of blood sampling and analysis, time of recanalization and reperfusion status, devices and technical management of EVT, peri-interventional and postinterventional complications, and short-term clinical outcome as assessed by the modified Rankin Scale (mRS) at hospital discharge.

### Statistical Analysis

Statistical analysis was performed using GraphPad Prism (GraphPad Prism 9.4.1, GraphPad Software, San Diego, CA, USA). Gaussian distribution of data was determined by the D’Agostino-Pearsons omnibus normality test. Numerical data were given as mean (95% confidence interval, CI) or median (interquartile range, IQR); categorical data were presented as number (percentage). Related samples were analyzed using Student’s t test or Wilcoxon signed-rank test, depending on parametric or nonparametric distribution of the data. Spearman’s rank correlation coefficient was applied to detect principal associations between ischemic ABG parameters and clinical-radiological variables. Post-hoc subgroup analyses by collateral status and infarct extent were conducted to further characterize relevant associations. A two-sided *P* value < 0.05 was considered statistically significant.

## Results

### Study Population

Between 18 December, 2018, and 31 August, 2020, *n* = 366 consecutive patients who were treated by EVT were assessed for study eligibility according to a prespecified study protocol [18–20]. *N* = 48 patients were excluded due to posterior circulation LVO and *n* = 11 patients due to bilateral or multifocal vessel occlusions. *N* = 50 patients did not qualify for inclusion upon invasive angiographic imaging either due to spontaneous recanalization or subocclusion with residual antegrade flow before EVT. Microcatheter-aspiration of ischemic blood samples was attempted in *n* = 257 patients with angiographically proven LVO of the following target sites: intracranial ICA‑T segment, MCA-M1, and proximal MCA-M2 segment, respectively. Microcatheter-aspiration of cerebral blood samples through blood gas syringes succeeded in *n* = 72 patients (28%). Out of this group, *n* = 51 consecutive patients (20%) met all a priori defined sampling and interventional criteria of inclusion and entered data analyses. The full patient flow without missing cases is given in Fig. 1. A detailed presentation of the ABG data including temporal, clinical functional and radiological structural correlations is given in Table 1, 2, 3, 4 and 5; a graphical summary is provided in the Supplementary Information.

**Table 1:** Patient characteristics

Variable		Study population *n* = 51
*Age, years, median (IQR)*		78 (69–83)
*Female sex, no. (%)*		34 (66.7)
**Cerebrovascular risk factors, no. (%)**		
*Arterial hypertension*		45 (88.2)
*Diabetes mellitus*		10 (19.6)
*Hyperlipidemia*		16 (31.4)
*Smoker status*		
Current		6 (11.8)
Former		8 (15.7)
Never		37 (72.5)
*Atrial fibrillation*		34 (66.7)
**Baseline drug treatment, no. (%)**		
*Antihypertensive drugs*		45 (88.2)
Antithrombotic drugs		13 (25.2)
Anticoagulation		20 (39.2)
**Clinical and radiological assessment at hospital admission**		
*Preprocedural blood pressure (mm* *Hg), median (IQR)*		
Systolic		161 (150–178)
Diastolic		89 (75–99)
*Heart rate, bpm, median (IQR)*		80 (71–96)
*NIHSS, median (IQR)*		13 (8–17)
*ASPECTS, median (IQR)*		9 (8–9)
*Unknown time of symptom onset, no. (%)*		17 (33.3)
**Acute stroke treatment**		
*Treatment with intravenous alteplase, no. (%)*		17 (33.3)
*Endovascular thrombectomy*		
*Occlusion location on invasive angiography, no. (%)*^*a*^		
Intracranial ICA		15 (29.4)
ICA‑T		7 (13.7)
MCA		
First segment, M1		19 (37.3)
Second segment, M2		17 (33.3)
*Right-hemispheric stroke*		19 (37.3)
*Collateral status on invasive angiographic series, median (IQR)*		2 (0–3)
*Procedural measures of EVT*		
Stroke onset-to-puncture time, minutes, median (IQR)		255 (181–353)
Imaging-to-groin puncture time, minutes, median (IQR)		45 (32–57)
Groin access-to-first device deployment time, minutes, median (IQR)		40 (32–49)
Number of thrombectomy passages, median (IQR)		2 (1–4)
Final eTICI score, no. (%)	0	2 (3.9)
	1	1 (2.0)
	2a (0–49%)	7 (13.7)
	2b50 (50–66%)	10 (19.6)
	2b67 (67–89%)	15 (29.4)
	2c (90–99%)	5 (9.8)
	3 (100%)	11 (21.6)
	Successful recanalization eTICI ≥ 2b50, no. (%)	41 (80.4)
Stroke onset-to-final recanalization time, minutes, median (IQR)		336 (274–424)
Stroke onset-to-ischemic sampling time, minutes, median (IQR)		274 (205–369)
Stroke onset-to-systemic sampling, minutes, median (IQR)		360 (310–458)
**Outcome**		
*ASPECTS at 24–48* *h after EVT, median (IQR)*		8 (6–9)
*Intracranial hemorrhage, no. (%)*		8 (15.7)
*NIHSS at 48* *h after EVT, median (IQR)*		9 (4–16)
*Modified Rankin Scale score at discharge, median (IQR)*		3 (1–4)
Modified Rankin Scale score of 0–2 at discharge, no. (%)		20 (39.2)
*In house mortality, no. (%)*		7 (13.7)

Data are presented as median (interquartile range) for continuous variables or as number (percentages) for categorical variables

*ASPECTS* Alberta Stroke Program Early CT Score, *bpm* beats per minute, *eTICI* extended thrombolysis in cerebral infarction scale, *EVT* endovascular thrombectomy, *ICA* internal carotid artery, *IQR* interquartile range, *MCA* *M1/M2* M1 or proximal M2 segment of the middle cerebral artery, *mmHg* millimeters of mercury, *NIHSS* National Institutes of Health Stroke Scale

^a^Including multiple occlusion locations

**Table 2:** ABG parameters during LVO stroke by the site of sampling

Parameter	Ischemic (95% CI)	Systemic (95% CI)	*P*	Normal range
pH	7.38 (7.37–7.4)	7.37 (7.35–7.38)	0.0019	7.36–7.45
*p*_a_CO_2_ (mm Hg)	36.94 (34.87–39.01)	38.62 (36.77–40.46)	0.16	32–46
*p*_a_O_2_ (mm Hg)	185.3 (160.5–210.1)	193.6 (166.8–220.3)	0.035	70–95
HCO_3_^−^std (mmol/L)	22.1 (21.02–23.17)	21.82 (20.98–22.66)	0.52	21–26
BE (B) (mmol/L)	−3.04 (−4.32–−1.76)	−3.28 (−4.27–−2.28)	0.64	−2–+3
ctCO_2_ (mmol/L)	22.27 (21.03–23.5)	22.4 (21.46–23.35)	0.85	23–27
Hct (%)	34.69 (32.70–36.68)	33.97 (31.87–36.06)	0.1	35–47
tHb (g/dl)	11.7 (11.04–12.37)	11.45 (10.75–12.15)	0.09	12–16
sO_2_ (%)	98.67 (98.23–99.11)	98.84 (98.48–99.21)	0.16	95–99
O_2_Hb (%)	97.88 (97.41–98.35)	98.09 (97.70–98.48)	0.19	96–100
COHb (%)	0.47 (0.36–0.57)	0.46 (0.34–0.58)	0.52	0–2
MetHb (%)	0.32 (0.24–0.41)	0.29 (0.21–0.38)	0.23	0–1.5
HHb (%)	1.34 (0.9–1.77)	1.17 (0.81–1.53)	0.14	0–5
Na^+^ (mmol/L)	138.5 (137.1–139.8)	137.6 (136.6–138.6)	0.11	135–145
K^+^ (mmol/L)	3.44 (3.27–3.59)	3.64 (3.44–3.85)	0.0083	3.6–5.2
Na^+^:K^+^ ratio	41.74 (38.38–45.1)	40.38 (36.07–44.69)	0.0048	27.9–37.5
iCa^2+^ (mmol/L)	1.11 (1.07–1.15)	1.12 (1.09–1.16)	0.26	1.15–1.25
Cl^−^ (mmol/L)	107.7 (105.9–109.6)	107.00 (105.1–108.8)	0.42	98–108
Glu (mg/dl)	122.3 (108.8–135.8)	122.00 (109.00–135.00)	0.1	55–100

All ABG data are given as mean value with 95% confidence interval (*CI*) and with normal ranges [31]

*ABG* arterial blood gas, *BE* *(B)* base deviation in plasma, *Cl*^*−*^ chloride ion concentration, *COHb* carboxyhemoglobin, *ctCO*_*2*_ total content of CO_2_, *HCO*_*3*_^*−*^ *std* standard bicarbonate, *Glu* glucose, *Hct* hematocrit, *HHb* deoxyhemoglobin, *iCa*^*2+*^ ionized calcium ion concentration, *K*^*+*^ potassium ion concentration, *MetHb* methemoglobin, *Na*^*+*^ sodium ion concentration, *Na*^*+*^*:K*^*+*^ *ratio* sodium-to-potassium ratio, *O*_*2*_*Hb* fractional oxyhemoglobin, *p*_*a*_*CO*_*2*_ arterial partial pressure of carbon dioxide, *p*_*a*_*O*_*2*_ arterial partial pressure of oxygen, *sO*_*2*_ oxygen saturation of hemoglobin, *tHb* total hemoglobin

**Table 3:** Correlation analysis between ischemic ABG parameters and duration of stroke

Ischemic parameter	r_s_ (95% CI)	*P*
pH	−0.36 (−0.65–0.02)	0.055
*p*_a_CO_2_ (mm Hg)	−0.17 (−0.51–0.22)	0.39
*p*_a_O_2_ (mm Hg)	−0.21 (−0.54–0.17)	0.27
HCO_3_^−^ std (mmol/L)	−0.34 (−0.63–0.05)	0.074
BE (B) (mmol/L)	−0.34 (−0.63–0.05)	0.074
ctCO_2_ (mmol/L)	−0.27 (−0.59–0.12)	0.16
Hct (%)	−0.14 (−0.28–0.52)	0.5
tHb (g/dl)	0.17 (−0.25–0.54)	0.4
sO_2_ (%)	−0.13 (−0.51–0.29)	0.53
O_2_Hb (%)	−0.03 (−0.43–0.38)	0.89
COHb (%)	0.06 (−0.35–0.46)	0.76
MetHb (%)	−0.21 (−0.57–0.21)	0.31
HHb (%)	0.13 (−0.29–0.51)	0.53
Na^+^ (mmol/L)	−0.19 (−0.52–0.18)	0.3
K^+^ (mmol/L)	0.12 (−0.25–0.47)	0.51
Na^+^:K^+^ ratio	0.14 (−0.16–0.42)	0.35
iCa^2+^ (mmol/L)	−0.18 (−0.51–0.21)	0.35
Cl^−^ (mmol/L)	−0.04 (−0.33–0.4)	0.83
Glu (mg/dl)	0.27 (−0.11–0.58)	0.15

All parameters were tested by Spearman’s rank correlation coefficient (*r*_*s*_) and are given with 95% confidence interval (*CI*). A two-sided *P* value of < 0.05 was used to determine statistical significance. *BE* *(B)* base deviation in plasma, *Cl*^−^ chloride ion concentration, *COHb* carboxyhemoglobin, *ctCO*_2_ total content of CO_2_, *HCO*_3_^−^ *std* standard bicarbonate, *Glu* glucose, *Hct* hematocrit, *HHb* deoxyhemoglobin, *iCa*^2+^ ionized calcium ion concentration, *K*^+^ potassium ion concentration, *MetHb* methemoglobin, *Na*^+^ sodium ion concentration, *Na*^+^*:K*^+^ ratio sodium-to-potassium ratio, *O*_2_*Hb* fractional oxyhemoglobin, *p*_a_*CO*_2_ arterial partial pressure of carbon dioxide, *p*_a_*O*_2_ arterial partial pressure of oxygen, *sO*_2_ oxygen saturation of hemoglobin, *tHb* total hemoglobin

**Table 4:** Correlation analysis of ischemic ABG parameters with baseline and follow-up NIHSS

Ischemic Parameter	NIHSS at admission		NIHSS at 48 h	
	r_s_ (95% CI)	*P*	r_s_ (95% CI)	*P*
pH	0.24 (−0.50–0.08)	0.12	0.01 (−0.31–0.32)	0.98
*p*_a_CO_2_ (mm Hg)	0.07 (−0.24–0.36)	0.67	−0.01 (−0.32–0.3)	0.95
*p*_a_O_2_ (mm Hg)	0.22 (−0.09–0.49)	0.16	−0.23 (−0.51–0.08)	0.13
HCO_3_^−^ std (mmol/L)	−0.07 (−0.37–0.24)	0.64	−0.05 (−0.35–0.27)	0.78
BE (B) (mmol/L)	−0.08 (−0.37–0.23)	0.61	−0.08 (−0.37–0.23)	0.61
ctCO_2_ (mmol/L)	−0.03 (−0.34–0.27)	0.83	−0.11 (−0.42–0.21)	0.48
Hct (%)	−0.11 (−0.43–0.24)	0.53	−0.08 (−0.4–0.27)	0.67
tHb (g/dl)	−0.17 (−0.48–0.18)	0.32	−0.16 (−0.48–0.2)	0.37
sO_2_ (%)	0.22 (−0.12–0.52)	0.19	−0.2 (−0.51–0.16)	0.26
O_2_Hb (%)	0.09 (−0.25–0.42)	0.59	−0.22 (−0.53–0.13)	0.2
COHb (%)	0.04 (−0.31–0.37)	0.84	0.22 (−0.14–0.53)	0.21
MetHb (%)	0.06 (−0.28–0.39)	0.71	0.12 (−0.24–0.45)	0.51
HHb (%)	−0.22 (−0.52–0.12)	0.19	0.2 (−0.16–0.51)	0.26
Na^+^ (mmol/L)	0.14 (−0.17–0.42)	0.37	−0.22 (−0.5–0.09)	0.16
K^+^ (mmol/L)	−0.07 (−0.35–0.23)	0.66	0.11 (−0.2–0.4)	0.49
Na^+^:K^+^ ratio	0.06 (−0.24–0.35)	0.69	−0.12 (−0.42–0.19)	0.43
iCa^2+^ (mmol/L)	−0.07 (−0.36–0.24)	0.66	−0.28 (−0.54–0.04)	0.08
Cl^−^ (mmol/L)	0.05 (−0.26–0.34)	0.76	−0.08 (−0.38–0.23)	0.61
Glu (mg/dl)	0.04 (−0.25–0.33)	0.77	0.37 (0.07–0.6)	0.015

All parameters were tested by Spearman’s rank correlation coefficient (*r*_*s*_) and are given with 95% confidence interval (*CI*). A two-sided *P* value of < 0.05 was used to determine statistical significance. *BE* *(B)* base deviation in plasma, *Cl*^−^ chloride ion concentration, *COHb* carboxyhemoglobin, *ctCO*_2_ total content of CO_2_, *HCO*_3_^−^ *std* standard bicarbonate, *Glu* glucose, *Hct* hematocrit, *HHb* deoxyhemoglobin, *iCa*^2+^ ionized calcium ion concentration, *K*^+^ potassium ion concentration, *MetHb* methemoglobin, *Na*^+^ sodium ion concentration, *Na*^+^*:K*^+^ ratio sodium-to-potassium ratio, *NIHSS* National Institutes of Health Stroke Scale, *O*_2_*Hb* fractional oxyhemoglobin, *p*_a_*CO*_2_ arterial partial pressure of carbon dioxide, *p*_a_*O*_2_ arterial partial pressure of oxygen, *sO*_2_ oxygen saturation of hemoglobin, *tHb* total hemoglobin

**Table 5:** Correlation analysis between ischemic ABG parameters and infarct extent before and after vessel recanalization

Ischemic Parameter	ASPECTS at admission		ASPECTS at 24–48 h		∆ ASPECTS	
	r_s_ (95% CI)	*P*	r_s_ (95% CI)	*P*	r_s_ (95% CI)	*P*
pH	0.18 (−0.13–0.46)	0.23	−0.02 (−0.32–0.29)	0.89	0.21 (−0.1–0.48)	0.17
*p*_a_CO_2_ (mm Hg)	0.15 (−0.16–0.44)	0.33	0.09 (−0.23–0.37)	0.61	0.1 (−0.21–0.39)	0.53
*p*_a_O_2_ (mm Hg)	−0.13 (−0.42–0.19)	0.41	−0.02 (−0.32–0.29)	0.9	−0.07 (−0.36–0.24)	0.67
HCO_3_^−^ std (mmol/L)	0.23 (−0.09–0.5)	0.14	0.02 (−0.29–0.32)	0.89	0.26 (−0.05–0.2)	0.09
BE (B) (mmol/L)	0.23 (−0.08–0.5)	0.14	0.02 (−0.28–0.33)	0.88	0.26 (−0.05–0.52)	0.09
ctCO_2_ (mmol/L)	0.21 (−0.1–0.49)	0.16	0.01 (−0.3–0.31)	0.97	0.27 (−0.04–0.53)	0.07
Hct (%)	0.18 (−0.17–0.49)	0.29	0.06 (−0.28–0.39)	0.72	0.1 (−0.25–0.42)	0.56
tHb (g/dl)	0.18 (−0.16–0.49)	0.28	0.07 (−0.26–0.4)	0.69	0.09 (−0.26–0.41)	0.62
sO_2_ (%)	−0.1 (−0.43–0.24)	0.54	0.04 (−0.3–0.37)	0.83	−0.21 (−0.51–0.13)	0.21
O_2_Hb (%)	−0.02 (−0.35–0.32)	0.93	0.1 (−0.25–0.42)	0.56	−0.15 (−0.46–0.2)	0.39
COHb (%)	0.04 (−0.37–0.31)	0.84	−0.04 (−0.37–0.3)	0.82	−0.12 (−0.44–0.23)	0.5
MetHb (%)	−0.2 (−0.51–0.15)	0.25	−0.11 (−0.43–0.24)	0.54	−0.06 (−0.39–0.28)	0.71
HHb (%)	0.11 (−0.24–0.43)	0.53	−0.04 (−0.37–0.31)	0.83	0.21 (−0.13–0.51)	0.21
Na^+^ (mmol/L)	−0.13 (−0.41–0.17)	0.39	−0.34 (−0.58–−0.05)	0.02	0.42 (0.14–0.64)	0.0033
K^+^ (mmol/L)	0.3 (0.00–0.55)	0.041	0.02 (−0.28–0.31)	0.91	0.24 (−0.06–0.5)	0.1
Na^+^:K^+^ ratio	−0.32 (−0.56–−0.02)	0.031	−0.09 (−0.38–0.22)	0.57	−0.15 (−0.43–0.16)	0.33
iCa^2+^ (mmol/L)	0.26 (−0.05–0.52)	0.09	0.01 (−0.3–0.31)	0.97	0.21 (−0.1–0.48)	0.17
Cl^−^ (mmol/L)	−0.35 (−0.59–−0.06)	0.018	−0.2 (−0.47–0.11)	0.19	−0.07 (−0.36–0.24)	0.66
Glu (mg/dl)	0.06 (−0.24–0.35)	0.69	0.04 (−0.26–0.33)	0.78	−0.0043 (−0.03–0.29)	0.98

All parameters were tested by Spearman’s rank correlation coefficient (*r*_*s*_) and are given with 95% confidence interval (*CI*). A two-sided *P* value of < 0.05 was used to determine statistical significance. *ASPECTS* Alberta Stroke Program Early CT Score, *BE* *(B)* base deviation in plasma, *Cl*^−^ chloride ion concentration, *COHb* carboxyhemoglobin, *ctCO*_2_ total content of CO_2_, *HCO3− std* standard bicarbonate, *Glu* glucose, *Hct* hematocrit, *HHb* deoxyhemoglobin, *iCa*^2+^ ionized calcium ion concentration, *K*^+^ potassium ion concentration, *MetHb* methemoglobin, *Na*^+^ sodium ion concentration, *Na*^+^*:K*^+^ ratio sodium-to-potassium ratio, *O*_2_*Hb* fractional oxyhemoglobin, *p*_a_*CO*_2_ arterial partial pressure of carbon dioxide, *p*_a_*O*_2_ arterial partial pressure of oxygen, *sO*_2_ oxygen saturation of hemoglobin, *tHb* total hemoglobin

### Patient Characteristics

The clinical, radiological, interventional, and sampling-related characteristics of the study population are presented in Table 1.

Among included patients, *n* = 34 (66.7%) were female and *n* = 17 (33.3%) were male. The median age of patients was 78 years (IQR 69–83 years). Arterial hypertension was the most prevalent comorbid disease (*n* = 45, 88.2%), followed by atrial fibrillation (*n* = 34, 66.7%). Hypercholesterolemia was found in *n* = 16 (31.4%) patients. Of the patients 10 (19.6%) had diabetes mellitus at the time of stroke diagnosis, 6 patients (11.8%) were current smokers, 8 patients (15.7%) were former smokers, and *n* = 37 patients (72.5%) had no smoking history. At the time of hospital admission, *n* = 45 (88.2%) patients received antihypertensive therapy, *n* = 13 (25.5%) patients received antiplatelet medication, and *n* = 20 (39.2%) patients received anticoagulant therapy. The median preprocedural systolic blood pressure was 161 mm Hg (IQR 150–178 mm Hg) and the median preprocedural diastolic blood pressure was 89 mm Hg (IQR 75–99 mm Hg). The median heart rate at baseline was 80 (IQR 71–96) beats per minute. Baseline clinical stroke severity as assessed by the NIHSS at hospital admission was 13 (IQR 8–17). The time of stroke onset was unknown in *n* = 17 patients (33.3%). Non-contrast computed tomography (NCCT) revealed a median baseline ASPECTS of 9 (IQR 8–9). A total of *N* = 34 (66.7%) patients were treated by EVT alone and *n* = 17 patients (33.3%) were treated by EVT with previous intravenous alteplase administration. The median time from symptom onset to groin puncture was 255 min (IQR 181–353 min). Invasive angiography confirmed the persistence of cerebral LVO in all patients and showed *n* = 15 (29.4%) intracranial ICA occlusions, *n* = 7 (13.7%) ICA‑T occlusions, *n* = 19 (37.3%) MCA-M1, and *n* = 17 (33.3%) MCA-M2 occlusions. The median invasive collateral score was 2 (IQR 0–3) [27]. Successful recanalization as assessed by the extended thrombolysis in cerebral infarction (eTICI) scale (eTICI ≥ 2b50) was achieved in 41 patients (80.4%) [28]. The median number of stent retriever passages required for recanalization was 2 (IQR 1–4). The median time interval from symptom onset to final recanalization was 336 min (IQR 274–462 min). No intraprocedural complications (e.g., thrombus fragmentation, vessel perforation or cerebral air embolism) were observed.

### Radiological and Early Clinical Outcome

Median ASPECTS on follow-up imaging at 48 h after intervention was 8 (IQR 6–9) [29]. Follow-up CT imaging revealed intracranial hemorrhages as defined by the Heidelberg bleeding classification (HBC) in eight patients (15.7%) [30], three patients (5.9%) showed petechial hemorrhages (HBC classes 1a and 1b), one patient (2%) developed a large space-occupying intraparenchymal hematoma (HBC class 2), and subarachnoid hemorrhages (HBC class 3c) were observed in two patients (3.9%). Clinical stroke severity at 48 h after recanalization was reflected by a median NIHSS score of 9 (IQR 4–16). The median modified Rankin Scale (mRS) score at hospital discharge was 3 (IQR 1–4). Functional independence (mRS ≤ 0–2) was achieved in *n* = 20 (39.2%) patients and *n* = 7 patients (13.7%) died during hospital stay. All other patients (*n* = 44, 86.3%) were discharged either home, to a rehabilitation facility or to another hospital.

### ABG Analysis in the Ischemic and Systemic Circulation

A total of *n* = 102 matched regional ischemic (intravascular sample from within the collateral circulation distal to the occlusion site) and systemic arterial blood samples (physiological flow conditions at cervical ICA level) were drawn from *n* = 51 LVO stroke patients. The data on ABG analysis of both sampling sites are presented in Table 2 and summarized in supplemental Fig. 1.

The following ABG parameters were examined: pH, *p*_a_CO_2_, *p*_a_O_2_, BE (B), HCO_3_^−^ std, and sO_2_. Significantly more alkaline conditions were present within collateral blood vessels (pH_ischemic_ = 7.38 vs. pH_systemic_ = 7.37; *p* = 0.0019). Concomitantly, the oxygen partial pressure was significantly decreased under vascular occlusion as compared to systemic level (*p*_a_O_2ischemic_ = 185.3 mm Hg vs. *p*_a_O_2systemic_ = 193.6 mm Hg; *p* = 0.035). By contrast, there were no sampling site-related differences in carbon dioxide partial pressure (*p*_a_CO_2ischemic_ = 36.94 mm Hg vs. *p*_a_CO_2 systemic_ = 38.62 mm Hg; *p* = 0.16), standard bicarbonate (HCO_3_^−^ std_ischemic_ = 22.1 mmol/L vs. HCO_3_^−^ std_systemic_ = 21.82 mmol/L; *p* = 0.52), and buffer base concentrations (BE_ischemic_ = −3.04 mmol/L vs. BE_systemic_ = −3.28 mmol/L; *p* = 0.64). Likewise, we found no differences in oxygen saturation of hemoglobin (sO_2ischemic_ = 98.67% vs. sO_2systemic_ = 98.84%; *p* = 0.16).

We assessed the following ion parameters: Na^+^ ion concentration, K^+^ ion concentration, Na^+^:K^+^ ratio, iCa^2+^ ion concentration and Cl^−^ ion concentration. The were no significant differences in sodium concentrations between the sampling sites (Na^+^_ischemic_ = 138.5 mmol/L vs. Na^+^_systemic_ = 137.6 mmol/L; *p* = 0.11). By contrast, potassium concentrations were significantly decreased within the hemodynamically compromised collateral circulation as compared to systemic levels (K^+^_ischemic_ = 3.44 mmol/L vs. K^+^_systemic_ = 3.64 mmol/L; *p* = 0.0083). Consistently, the regional ischemic Na^+^:K^+^ ratios were significantly increased (Na^+^:K^+^ ratio_ischemic_ = 41.74 vs. Na^+^:K^+^ ratio_systemic_ = 40.38; *p* = 0.0048). There were no significant differences between ischemic and systemic ionized calcium (iCa^2+^_ischemic_ = 1.11 mmol/L vs. iCa^2+^_systemic_ = 1.12 mmol/L; *p* = 0.26) and chloride concentrations (Cl^−^_ischemic_ = 107.7 mmol/L vs. Cl^−^_systemic_ = 107.00 mmol/L; *p* = 0.42).

### Association Between Ischemic Blood Gas Parameters and the Duration of Stroke

We used Spearman’s rank correlation coefficient to assess associations between ischemic ABG parameters and the time from stroke onset to ischemic sampling. The median time from stroke onset to distal sampling was 274 min (IQR 205–369 min). Regional ischemic pH values were found to be negatively associated with the duration of stroke, but the correlation was just above the threshold for statistical significance (r = −0.36; *p* = 0.055). There was also a borderline statistical insignificance for the association of local HCO_3_^−^ std (r = −0.34; *p* = 0.074) and BE (B) (r = −0.34; *p* = 0.074) with occlusion duration. All other ischemic ABG parameters showed no correlation with the time from stroke onset to ischemic sampling (|r| < 0.3; Table 3 and supplemental Fig. 1).

### Association of Ischemic ABG Parameters with Clinical Stroke Severity Before and After Vessel Recanalization

Spearman’s rank correlation coefficient was used to investigate associations between the ischemic ABG parameters and clinical stroke severity at baseline and at 48 h after recanalization. These data are given in Table 4 and supplemental Fig. 1. Blood glucose levels within the collateral circulation were positively correlated with the NIHSS score at 48 h after recanalization (r = 0.37; *p* = 0.015); however, none of the other ischemic ABG parameters showed any association with either clinical stroke severity on admission or at 48 h.

### Association of Ischemic ABG Parameters with Infarct Extent Before and After Vessel Recanalization

Results of correlation analysis between regional ischemic ABG parameters and infarct extent before and after EVT are given in Table 5 and supplemental Fig. 1. Univariate analysis revealed significant associations between preserved tissue integrity (ASPECTS) at admission and intravascular Na^+^:K^+^ ratios (r = −0.32; *p* = 0.031), K^+^ concentrations (r = 0.3; *p* = 0.041), and Cl^−^ concentrations (r = −0.35; *p* = 0.018) distal to the occlusion site. No other associations between baseline infarct extent and ischemic ABG parameters were seen. Analysis of dichotomized data (more extensive infarcts, ASPECTS ≤ 7 vs. minor infarcts, ASPECTS ≥ 8; poor vs. moderate to good collaterals) did not show a difference between any of the ischemic ABG parameters (Supplementary Information).The Na^+^ concentrations within collateral blood vessels were found to be negatively correlated with postinterventional infarct extent 24–48 h after recanalization (r = −0.34, 95% CI: −0.58–−0.05; *p* = 0.02). There were no other correlations between the ischemic ABG parameters and tissue integrity on follow-up imaging.

We further analyzed the association between imaging defined preinterventional to postinterventional worsening of stroke (∆ ASPECTS) and regional ischemic ABG parameters. Infarct extent remained constant in *n* = 20 (39.2%) patients, whereas dynamic infarct progression equivalent to a median ∆ ASPECTS of 1 (IQR 1–2) was observed in *n* = 31 (60.8%) patients. Progressive infarction was positively correlated with the Na^+^ concentrations within the occluded vascular territory (r = 0.42; *p* = 0.0033). No other associations were found with respect to preinterventional to postinterventional infarct progression.

## Discussion

To the best of our knowledge, no dedicated animal and only few preliminary human reports exist on regional disturbances of blood gas and ion homeostasis measured directly within the cerebral collateral circulation during acute ischemic stroke [14–16, 32]. This represents an observation gap to understand stroke pathophysiology, because pioneering experimental observations have highlighted the relevance of low blood flow and metabolic failure including tissue ion shifts particularly during the initial phase of infarct formation; however, without providing data on the cerebral vascular compartment [5, 6]. Recently, we have developed a method which enables the noise-controlled extraction and analysis of cerebral blood samples which were obtained from collateral arteries under persistent ischemic conditions [18, 19]. This approach proved to be highly consistent across different research designs [18–23, 33] and was now used to investigate the clinical significance of cerebral blood gas and electrolyte alterations in human LVO stroke.

Our main findings are the following: human LVO stroke results in a (1) regional reduction in oxygen partial pressure (−4.29%; *p* = 0.035) and (2) potassium concentrations (−5.49%; *p* = 0.0081) within the collateral circulation. (3) The regional Na^+^:K^+^ ratio is increased and (4) correlated with the extent of ischemic brain edema as assessed by the ASPECTS system (r = −0.32, *p* = 0.031). (5) Regional intravascular sodium release is correlated with infarct progression after recanalization (r = 0.42, *p* = 0.0033). Finally, (6) more alkaline conditions are present within the arterial compartment of the ischemic penumbra (+0.14%; *p* = 0.0019) which change (7) towards more acidotic pH values over time (r = −0.36, *p* = 0.055).

The relative reduction in oxygen partial pressure distal to the occlusion site is consistent with previous observations which, however, found oxygen partial pressure both within (*p*_a_O_2ischemic_ = 73.90 mm Hg vs. *p*_a_O_2systemic_ = 78.90 mm Hg) and well above (*p*_a_O_2ischemic_ = 213.98 mm Hg vs. *p*_a_O_2systemic_ = 251.43 mm Hg) the limits for arterial blood [14, 15]. Substantial elevations in *p*_a_O_2_, as were observed in our study, are explained by preinterventional and peri-interventional high-flow oxygenation which is known to lead to an up to fourfold increase in *p*_a_O_2_ [34]. In our study, disruption of antegrade cerebral blood flow led to regional normocapnia and negative base excess. This condition plausibly reflects an incipient metabolic (nonrespiratory) acidosis [35]. At this time of observation, full compensation, as reported previously [15, 16], is unlikely as counterregulation is not accomplished within 12 h [36, 37]. Interestingly, ABG analysis revealed both a fine but significant shift to more alkaline overall pH values within collateral blood vessels and a time-dependent decrease in cerebral pH values during occlusive ischemia. As more pronounced acidosis, reflecting impaired CO_2_ removal during no/low-flow conditions or inadequate oxidative phosphorylation, is to be expected in or in close vicinity to the infarct core, it can be inferred that cerebral samples were extracted from penumbral regions where an infarct milieu is gradually developing [13, 38–40]. This interpretation is supported by the favorable imaging profile of the study population which is characterized by predominantly small baseline infarcts (i.e., median ASPECTS of 9 and penumbral imaging-based patient selection). Importantly, the initial ASPECTS in this study is numerically identical to that of pooled patient data from EVT trials in intermediate time windows (HERMES meta-analysis: 9) [25], and one point higher compared to that of both pooled patient data from EVT trials in extended time windows (AURORA meta-analysis: 8) and large prospective registries (German stroke registry: 8 and STRATIS registry: 8) [1, 41, 42]. Hence, our observations may also apply to these populations and are likely not driven by a selection bias due to overrepresentation of patients with small ischemic lesions.

Normal neuroelectric activity and water content of the brain require the careful orchestration and proper distribution of intracellular and extracellular ions. The observed relative hypokalemia and increase in Na^+^/K^+^ ratios support the notion of significant ion movements within ischemic brain regions which are characterized by net K^+^ losses and/or Na^+^ gains [6, 7]. The literature suggests that astrocytes may form a functional syncytium for extracellular and intravascular potassium clearance as a means to control neuronal excitability in viable tissue [7]. Correspondingly, the lack of massive sodium release supports the conclusion that large-scale cell death may not have occurred in EVT patients at the time of cerebral sample acquisition [6, 43]. This is again consistent with the fact that more than one third of patients presenting with unknown or extended time of ischemia were selected for recanalization based on the absence of extensive infarction on imaging at presentation. Accordingly, there was no temporal association between cerebral ion composition and the duration of stroke. Finally, preclinical data from others indicate that there is a near-perfect linear correlation between sodium and potassium ion shifts and changes in brain water content in the lesioned hemisphere early before the disruption of the blood-brain barrier (r = 0.992; *p* < 0.001) [43]. Given that brain water content is inversely correlated with X‑ray attenuation, the results imply that the radiological measures of ASPECTS and ASPECTS decline reflect the extent and dynamics of ionic brain edema [29, 43, 44].

The major strength of this study is its prospective consecutive design including control of a large set of baseline clinical, radiological, interventional, and analytical variables. Still, this study remains observational and could be carried out only at a single-center limiting causal inference and generalizability. Furthermore, additional information regarding cerebral sodium content and pH based on, e.g., sodium and chemical exchange saturation transfer (CEST) magnetic resonance imaging (MRI) is not available [45, 46]; however, such a type of extended and methodically demanding MRI set-up would be highly time-consuming and this would represent a relevant time conflict with significant delay before EVT [24, 45, 46].

In conclusion, subtle but distinct disturbances of cerebral oxygen supply, ion composition and acid-base balance occur and dynamically progress during the phase of occlusive ischemia in stroke patients and are related to acute tissue damage. Combined, these data additionally advocate to restore physiologic cerebral circulatory conditions in LVO stroke, notwithstanding that promising new concepts of add-on cerebroprotection are emerging.

### Supplementary Information

Supplementary Figure 1 (sketch of the procedure and graphical representation of the main results); Supplementary Tables 1 and 2 (subgroup analyses by collateral status and infarct extent).
