# Supplementary material for: Distinct Alterations in Oxygenation, Ion Composition and Acid-Base Balance in Cerebral Collaterals During Large-Vessel Occlusion Stroke
Source: Clin Neuroradiol. 2023 Jun 7;33(4):973–84. doi: 10.1007/s00062-023-01296-w (PMC10654170; doi:10.1007/s00062-023-01296-w)
Supplement: Supplementary file 1 — Supplementary Figure 1 (sketch of the procedure and graphical representation of the main results); Supplementary Tables 1 and 2 (subgroup analyses by collateral status and infarct extent). [file 62_2023_1296_MOESM1_ESM.docx]

Supplementary Information

**Distinct Alterations in Oxygenation, Ion Composition and Acid-Base Balance in Cerebral Collaterals During Large-Vessel Occlusion Stroke**

Jörn Feick, Mirko Pham, Alexander G. März, Marius L. Vogt, Marc Strinitz, Guido Stoll, Michael K. Schuhmann, and Alexander M. Kollikowski

**Affiliations**

Jörn Feick, ORCID: 0000-0003-1187-0119

Department of Neuroradiology, University Hospital Würzburg, Würzburg, Germany

Mirko Pham, ORCID: 0000-0003-1295-2685

Department of Neuroradiology, University Hospital Würzburg, Würzburg, Germany

Alexander G. März, ORCID: 0000-0002-1358-0871

Department of Neuroradiology, University Hospital Würzburg, Würzburg, Germany

Marius L. Vogt, ORCID: 0000-0002-8297-1614

Department of Neuroradiology, University Hospital Würzburg, Würzburg, Germany

Marc Strinitz, ORCID: 0000-0002-2581-2000

Department of Neuroradiology, Klinikum rechts der Isar, Technical University Munich, Munich, Germany

Guido Stoll, ORCID: 0000-0003-2474-5165

Department of Neurology, University Hospital Würzburg, Würzburg, Germany

Michael K. Schuhmann, ORCID: 0000-0002-4014-7010

Department of Neurology, University Hospital Würzburg, Würzburg, Germany

Alexander M. Kollikowski (corresponding author), ORCID: 0000-0002-0288-5928

E-Mail: kollikowsk_a@ukw.de

Department of Neuroradiology, University Hospital Würzburg, Würzburg, Germany

**Figure 1:** Cerebral arterial blood sampling, regionally differing ABG parameters, and pathophysiological associations of ischemia-induced ABG alterations **
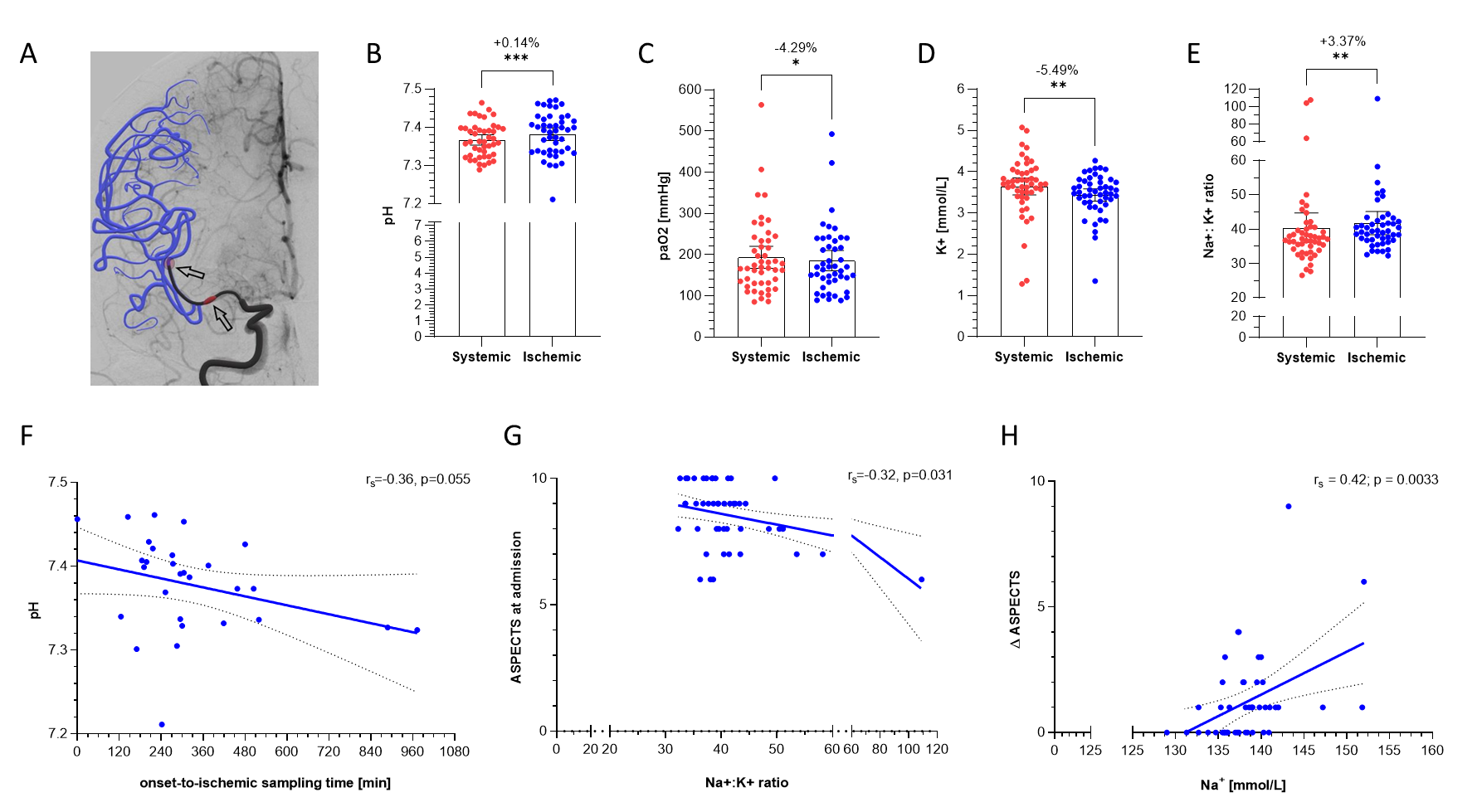
**

A: Direct sampling from collateral blood vessels (target region: ischemic territory, blue) during total occlusion (embolus, red) of the M1 segment of the middle cerebral artery, micro- (distal to occlusive lesion), and intermediate catheter (proximal to occlusive lesion) positioned as intended (black). B-D: Arterial blood gas (ABG) analysis including pH, arterial partial pressure of oxygen (p_a_O_2_), potassium (K^+^) ion concentration, and sodium-to-potassium (Na^+^:K^+^) ratio. Each dot represents related systemic versus ischemic blood samples during acute human stroke (n=51). Scatter dot plot with mean and 95% confidence interval (CI). Wilcoxon matched-pairs signed-rank test or Student’s t test; asterixis indicate significance (*P<0.05, **P<0.01, ***P<0.001). F: Linear correlation between ischemic pH (y-axis) and duration of stroke (onset-to-ischemic sampling time, x-axis). Spearman rank order correlation. G: Linear correlation between Alberta Stroke Program Early CT Score (ASPECTS) at admission (y-axis) and Na^+^:K^+^ ratio (x-axis). Spearman rank order correlation. H: Linear correlation between infarct progression after recanalization (ΔASPECTS, y-axis) and ischemic Na+ ion concentration (x-axis). Spearman rank order correlation.

**Table 1:** ABG parameters during large-vessel occlusion stroke by collateral status

| **Ischemic Parameter** | **Poor collaterals**  **(95% CI)** | **Moderate to good collaterals**  **(95% CI)** | **P** |
| --- | --- | --- | --- |
| pH | 7.38  (7.34 – 7.41) | 7.39  (7.37 – 7.41) | 0.61 |
| p_a_CO_2_ (mmHg) | 38.86  (35.68 – 42.05) | 35.59  (32.95 – 38.23) | 0.12 |
| p_a_O_2_ (mmHg) | 189  (160.5 – 217.6) | 185.6  (149.4 – 221.8) | 0.47 |
| HCO_3_^-^std (mmol/L) | 23.01  (20.79 – 25.23) | 21.62  (20.49 – 22.76) | 0.2 |
| BE (B) (mmol/L) | -2.09  (-4.7 – -0.51) | -3.52  (-4.91 – -2.13) | 0.28 |
| ctCO_2_ (mmol/L) | 22.98  (20.77 – 25.18) | 21.88  (20.38 – 23.37) | 0.38 |
| Hct (%) | 35.89  (32.28 – 39.5) | 34.38  (31.91 – 36.85) | 0.52 |
| tHb (g/dl) | 12  (10.77 – 13.23) | 11.65  (10.82 – 12.47) | 0.64 |
| sO_2_ (%) | 98.8  (97.72 – 99.88) | 98.65  (98.2 – 99.1) | 0.45 |
| O_2_Hb (%) | 98.13  (97.1 – 99.17) | 97.88  (97.35 – 98.4) | 0.47 |
| COHb (%) | 0.35  (0.13 – 0.57) | 0.47  (0.36 – 0.57) | 0.11 |
| MetHb (%) | 0.31  (0.17 – 0.45) | 0.31  (0.2 – 0.42) | 0.92 |
| HHb (%) | 1.23  (0.17 – 2.29) | 1.35  (0.9 – 1.8) | 0.45 |
| Na^+^ (mmol/L) | 139.0  (136.6 – 141.5) | 138.1  (136.6 – 139.6) | 0.2 |
| K^+^ (mmol/L) | 3.52  (3.27 – 3.77) | 3.42  (3.22 – 3.61) | 0.35 |
| Na^+^: K^+^ ratio | 40.21  (36.44 – 43.99) | 42.23  (37.52 – 46.95) | 0.37 |
| iCa^2+^ (mmol/L) | 1.14  (1.06 – 1.22) | 1.09  (1.05 – 1.14) | 0.11 |
| Cl^-^ (mmol/L) | 107.4  (104.7 – 110.1) | 107.8  (105.3 – 110.3) | 0.52 |
| Glu (mg/dl) | 117.7  (94.94 – 140.5) | 124  (106.9 – 141) | 0.6 |

Arterial phase CT-angiography collaterals at baseline were categorized as poor (n=17) or moderate to good (n=33, combined) based on the Miteff et al. grading scale. All ABG data are given as mean value with 95% confidence interval (CI). P values are based on the Wilcoxon matched-pairs signed-rank test or Student’s t test, as appropriate.

ABG, arterial blood gas; BE (B), base deviation in plasma; Cl^-^, chloride ion concentration; COHb, carboxyhemoglobin; ctCO_2_, total content of CO_2_; HCO_3_^-^ std, standard bicarbonate; Glu, glucose; Hct, hematocrit; HHb, deoxyhemoglobin; iCa^2+^, ionized calcium ion concentration; K^+^, potassium ion concentration; MetHb, methemoglobin; Na^+^, sodium ion concentration; Na^+^:K^+^ ratio, sodium-to-potassium ratio; O_2_Hb, fractional oxyhemoglobin; p_a_CO_2_, arterial partial pressure of carbon dioxide; p_a_O_2_, arterial partial pressure of oxygen; sO_2,_ oxygen saturation of hemoglobin; and tHb, total hemoglobin.

**Table 2:** ABG parameters during large-vessel occlusion stroke by infarct extent on baseline imaging

| **Ischemic Parameter** | **ASPECTS at admission** **≤7**  **(95% CI)** | **ASPECTS at admission ≥8**  **(95% CI)** | **P** |
| --- | --- | --- | --- |
| pH | 7.38  (7.34 – 7.41) | 7.39  (7.37 – 7.41) | 0.45 |
| p_a_CO_2_ (mmHg) | 35.44  (30.38 – 40.5) | 37.36  (35.19 – 39.53) | 0.4 |
| p_a_O_2_ (mmHg) | 180.7  (143 – 218.4) | 187.2  (156.7 – 217.7) | 0.9 |
| HCO_3_^-^std (mmol/L) | 21.28  (18.3 – 24.25) | 22.44  (21.4 – 23.48) | 0.33 |
| BE (B) (mmol/L) | -4.04  (-7.67 – -0.42) | -2.62  (-3.84 – -1.4) | 0.31 |
| ctCO_2_ (mmol/L) | 21.18  (17.74 – 24.63) | 22.7  (21.53 – 23.88) | 0.26 |
| Hct (%) | 34.19  (28.75 – 39.63) | 35  (33.11 – 37.14) | 0.84 |
| tHb (g/dl) | 11.52  (9.73 – 13.31) | 11.86  (11.18 – 12.54) | 0.77 |
| sO_2_ (%) | 98.84  (98.29 – 99.39) | 99.63  (98.7 – 99.4) | 0.99 |
| O_2_Hb (%) | 98.05  (97.37 – 98.73) | 97.88  (97.3 – 98.46) | 0.79 |
| COHb (%) | 0.53  (0.23 – 0.83) | 0.43  (0.34 – 0.53) | 0.93 |
| MetHb (%) | 0.29  (0.13 – 0.45) | 0.32  (0.22 – 0.42) | 0.72 |
| HHb (%) | 1.16  (0.61 – 1.7) | 1.38  (0.84 – 1.92) | 0.99 |
| Na^+^ (mmol/L) | 140.2  (137.1 – 143.2) | 137.8  (136.5 – 139.2) | 0.21 |
| K^+^ (mmol/L) | 3.25  (2.76 – 3.75) | 3.52  (3.39 – 3.65) | 0.59 |
| Na^+^: K^+^ ratio | 47.33  (34.18 – 60.49) | 39.61  (37.99 – 41.22) | 0.4 |
| iCa^2+^ (mmol/L) | 1.06  (0.94 – 1.18) | 1.13  (1.1 – 1.16) | 0.82 |
| Cl^-^ (mmol/L) | 110.6  (104.1 – 117.1) | 106.6  (105.3 – 107.9) | 0.32 |
| Glu (mg/dl) | 118.8  (85.07 – 152.6) | 122.9  (108.6 – 137.3) | 0.88 |

The Alberta Stroke Program Early CT Score (ASPECTS) at baseline was dichotomized into ≤7 (n=12) and ≥8 (n=39). All ABG data are given as mean value with 95% confidence interval (CI). P values are based on the Wilcoxon matched-pairs signed-rank test or Student’s t test, as appropriate.

ABG, arterial blood gas; BE (B), base deviation in plasma; Cl^-^, chloride ion concentration; COHb, carboxyhemoglobin; ctCO_2_, total content of CO_2_; HCO_3_^-^ std, standard bicarbonate; Glu, glucose; Hct, hematocrit; HHb, deoxyhemoglobin; iCa^2+^, ionized calcium ion concentration; K^+^, potassium ion concentration; MetHb, methemoglobin; Na^+^, sodium ion concentration; Na^+^:K^+^ ratio, sodium-to-potassium ratio; O_2_Hb, fractional oxyhemoglobin; p_a_CO_2_, arterial partial pressure of carbon dioxide; p_a_O_2_, arterial partial pressure of oxygen; sO_2,_ oxygen saturation of hemoglobin; and tHb, total hemoglobin.
